# Development of a copper metabolism-related gene signature in lung adenocarcinoma

**DOI:** 10.3389/fimmu.2022.1040668

**Published:** 2022-11-29

**Authors:** Wuguang Chang, Hongmu Li, Leqi Zhong, Tengfei Zhu, Zenghao Chang, Wei Ou, Siyu Wang

**Affiliations:** Department of Thoracic Surgery, Sun Yat-sen University Cancer Center, State Key Laboratory of Oncology in South China, Guangzhou, China

**Keywords:** copper, copper metabolism-related gene, prognosis, tumor microenvironment, immunotherapy, biomarker

## Abstract

**Purpose:**

The dysregulation of copper metabolism is closely related to the occurrence and progression of cancer. This study aims to investigate the prognostic value of copper metabolism-related genes (CMRGs) in lung adenocarcinoma (LUAD) and its characterization in the tumor microenvironment (TME).

**Methods:**

The differentially expressed CMRGs were identified in The Cancer Genome Atlas (TCGA) of LUAD. The least absolute shrinkage and selection operator regression (LASSO) and multivariate Cox regression analysis were used to establish the copper metabolism-related gene signature (CMRGs), which was also validated in Gene Expression Omnibus (GEO) database (GSE72094). The expression of key genes was verified by quantitative real-time PCR (qRT-PCR). Then, the CMRGS was used to develop a nomogram to predict the 1-year, 3-year, and 5-year overall survival (OS). In addition, differences in tumor mutation burden (TMB), biological characteristics and immune cell infiltration between high-risk and low-risk groups were systematically analyzed. Immunophenoscore (IPS) and an anti-PD-L1 immunotherapy cohort (IMvigor210) were used to verify whether CMRGS can predict the response to immunotherapy in LUAD.

**Results:**

34 differentially expressed CMRGs were identified in the TCGA dataset, 11 of which were associated with OS. The CMRGS composed of 3 key genes (LOXL2, SLC31A2 and SOD3) had showed good clinical value and stratification ability in the prognostic assessment of LUAD patients. The results of qRT-PCR confirmed the expression of key CMRGs in LUAD and normal tissues. Then, all LUAD patients were divided into low-risk and high-risk groups based on median risk score. Those in the low-risk group had a significantly longer OS than those in the high-risk group (P<0.0001). The area under curve (AUC) values of the nomogram at 1, 3, and 5 years were 0.734, 0.735, and 0.720, respectively. Calibration curves comparing predicted and actual OS were close to ideal model, indicating a good consistency between prediction and actual observation. Functional enrichment analysis showed that the low-risk group was enriched in a large number of immune pathways. The results of immune infiltration analysis also confirmed that there were a variety of immune cell infiltration in the low-risk group. In addition, multiple immune checkpoints were highly expressed in the low-risk group and may benefit better from immunotherapy.

**Conclusion:**

CMRGS is a promising biomarker to assess the prognosis of LUAD patients and may be serve as a guidance on immunotherapy.

## Introduction

Lung cancer is the most common type of cancer around the world, and the leading cause of cancer related death (1). About 85% of lung cancer cases are non-small cell lung cancer (NSCLC), the most common of which is lung adenocarcinoma (LUAD) (2). Although significant progress has been made in the field of molecular targeted therapy and immunotherapy in recent years, only part of patients can benefit from it (3, 4), and the 5-year survival rate for lung cancer is only 19% (5). Therefore, there is an urgent need to develop novel and effective prognostic markers to improve survival in LUAD patients.

The essential micronutrient copper plays a key role in a variety of biological processes, including mitochondrial respiration, antioxidant defense, and biocompound synthesis, and dysregulation of copper homeostasis can cause oxidative stress and cytotoxicity (6). Growing evidence suggests that copper plays an important role in cancer proliferation, angiogenesis and metastasis (7). The rapid proliferation of tumors requires a lot of energy. As a key cofactor of mitochondrial cytochrome C, copper is an important link in energy metabolism. Therefore, copper is increased in cancer tissues compared to healthy tissues (8). Serum and tumor tissue levels of copper have been found to be significantly elevated in patients with various cancers (lung, breast, colorectal, prostate, thyroid, and brain tumors) (9–14). Cancer cells require a large amount of oxygen from mitochondria for their proliferation, and excess copper can disrupt mitochondrial respiration, thereby inhibiting tumor growth (15). Furthermore, the antitumor effect of the immune system depends on intact mitochondrial metabolism (16), and the imbalance of copper will lead to the decline of immune response to tumor cells. Therefore, the signature of CMRGs may serve as a promising predictor for assessing prognosis and immunotherapy response in LUAD.

In this study, we constructed CMRGS containing LOXL2, SLC31A2 and SOD3 by the LUAD dataset in the TCGA database. This signature could effectively predict the prognosis of LUAD patients. We then further explored the correlation between CMRGS and TME. The results showed that the low-risk group was in a state of immune activation and thus had a large number of immune cell infiltration. Prediction of immunotherapy response also suggested that low-risk group could benefit better from immunotherapy. Overall, this study found that CMRGS can help predict the prognosis of patients with LUAD and may guide immunotherapy.

## Material and methods

### Data collection

The RNA-seq dataset of the TCGA-LUAD cohort with complete clinical information was downloaded from The Cancer Genome Atlas (TCGA) while excluding patients with missing survival information. Both Read counts and Fragments per Kilobase Million (FPKM) were collected. The dataset GSE72094 of 398 LUAD patients with complete clinical information was downloaded from the Gene Expression Omnibus (GEO). The flowchart of this study was shown in the **Figure S1**.

### Differentially expressed CMRGs

Copper metabolism-related genes were downloaded from Molecular Signatures Database v7.5.1 (MsigDB) (**Table S1**) (17). The “limma” package was used to identify differentially expressed CMRGs between LUAD and normal tissues (18). Thresholds were set as false discovery rate (FDR) < 0.05 and log2 |fold change| ≥ 1.

### Construction and validation of CMRGS

First, univariate cox regression analysis was performed on the differentially expressed CMRGs. The LASSO Cox regression algorithm was subsequently performed on OS-related CMRGs (p < 0.05) to further eliminate overfitting by “glmnet” package. Finally, CMRGS was constructed by multivariate stepwise Cox regression. Risk score for each patient=β_gene1_*Exp_gene1_+β_gene2_*Exp_gene2_ + ⋯ +β_genen_*Exp_genen_. Patients were divided into high- or low-risk groups based on the median risk score. Kaplan-Meier survival analysis and log-rank test compared the OS difference between the two groups. 398 LUAD case from GSE72094 were used to validate CMRGS. The same formula was used to calculate the risk score for each patient, and KM survival analysis was performed by “survival” and “survminer” package. Subgroups of different clinical characteristics were then subjected to stratified survival analysis to determine whether the characteristics were influenced by these factors.

### Transcription factors regulatory network and GO/KEGG enrichment analysis

The Citome Data Browser (DB) is an online website for analyzing the regulation of transcription factors (19) (http://cistrome.org/db/#/). In order to explore the potential mechanism and pathway of key CMRGs, we used the Cistrome database to query relevant transcription factors, and only differentially expressed transcription factors (FDR <0.05, log2 |fold change| ≥ 1) would be contained. Subsequently, we conducted GO and KEGG analysis on these key CMRGs and transcription factors based on “clusterprofiler” package (20).

### Establishment of the nomogram

To provide clinicians with a quantitative method for predicting the prognosis of patients with LUAD, we constructed the nomogram based on traditional clinical features and CMRGS to predict the 1, 3, and 5-year survival rates of patients. Univariate Cox analysis was first performed. Variables with P < 0.05 were subsequently included in multivariate Cox regression. Finally, the nomogram was constructed according to the results of multivariate Cox regression analysis by “rms” package. Accuracy of the model was evaluated by calibration curve and receiver operating characteristic (ROC) to determine the prognostic value.

### Analysis of TMB

Somatic mutation data of LUAD patients were downloaded from TCGA database. According to the total number of somatic mutations per MB base in the exon coding region of the human genome, the TMB of each LUAD sample is calculated as the total number of somatic mutations (including nonsynonymous point mutations, insertions and deletions in the exon coding region)/35MB. Subsequently, the somatic mutations of the high- and low-risk score groups were visualized using the “maftools” package. And the correlation between risk score and TMB was analyzed.

### Gene set enrichment analysis (GSEA)

In order to study the differences in biological function between high- and low-risk groups, GSEA (Version 4.2.3; http://software.broadinstitute.org/gsea/index.jsp) was conducted between two groups with GO and KEGG (21). “c5.go.v7.5.1.symbols” and “c2.cp.kegg.v7.5.1.symbols” were downloaded from MSigDB database. The threshold was set at P < 0.05 and FDR < 0.25.

### Analysis of TME cell infiltration in LUAD

Single gene set enrichment analysis (ssGSEA) was used to evaluate the scores of 28 kinds of immune cells in each LUAD patients by “GSVA” package (22).Then, the tumor purity and TME score (including ESTIMATE score, immune score and stromal score) of each patient were estimated using the “estimate” package (23).

### Prediction of immunotherapy response

To explore the potential impact of CMRGS on immunotherapy response, we analyzed the expression differences of key immune checkpoints between the two subgroups. IPS is an effective predictor of response to immunotherapy targeting CTLA-4 and PD-1 (24). The imvigor210 cohort is a group of patients with locally advanced and metastatic urothelial cancer who received anti-PD-L1 immunotherapy (http://research-pub.gene.com/IMvigor210CoreBiologies/packageVersions/) (25). IPS and imvigor210 were used to predict the response of the two subgroups to immunotherapy.

### Quantitative real-time PCR (qRT-PCR)

The expression profiles were examined by qRT-PCR. This study was approved by the Sun Yat-sen University Cancer Center ethics committee (YB2018-85). The samples were derived from patients who had undergone lung radical surgery at our center. Total RNA was isolated from LUAD tissue samples and the adjacent normal tissue samples using the TRIzol (TIANGEN, Beijing, China) and reverse-transcribed to complementary DNA using PrimeScript™ RT Master Mix (ES Science, Shanghai, China). Three hub gene was amplified using qRT-PCR by SYBR Green Master Mix (ES Science, Shanghai, China). The PCR primers used for amplification were as follows: SOD3,5′- TATACCGAGACCCACCATCCTT-3′(forward),5′- TTTCGGTACAAATGGAGGCCTT-3′(reverse); LOXL2,5′- GGCTCTTAAACAACCAGCTGTC-3′(forward),5′- TCGTTCAGACTCAGTTGTTGGG -3′(reverse); SLC31A2,5’-CAGGCATGGTCTTGTGTCTTAA-3’ (forward),5′- TCTCCCTGGCTTGAAATCTTTG-3′(reverse); GAPDH as an endogenous control, 5′-ATCAAGAAGGTGGTGAAGCAGG-3′(forward),5′-CGTCAAAGGTGGAGGAGTGG-3′ (reverse). The relative expression levels of UPF1 were calculated using the 2 − ΔΔCT method.

### Statistical analysis

Wilcoxon test was used to analyze the differences between the two groups. Kaplan Meier (KM) analysis and log rank test were applied to survival assessment. Student t-test was used to analyze the expression of key genes in tumor and normal tissues. Establishing risk factors for LUAD by cox proportional hazards regression analysis. Correlation analysis took Spearman’s rank correlation. P < 0.05 was considered statistically significant. All statistical analyses were performed using SPSS (version 26.0) and R 4.1.3 (https://www.r-project.org).

## Results

### Construction and evaluation of CMRG signature

**Figure 1:**
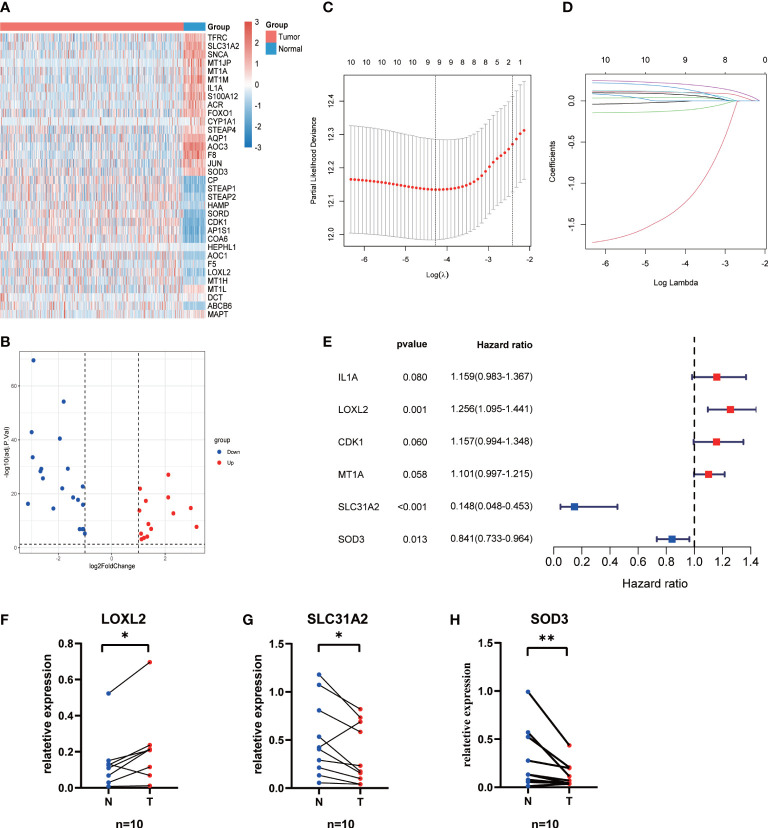
Construction of the CMRGS in LUAD. **(A)** Heatmap showing expression level of differentially expressed CMRGs. **(B)** Volcano plot exhibiting 20 down-regulated and 13 up-regulated CMRGs. **(C, D)** Lasso Cox regression analysis of 11 prognosis-related CMRGs. **(E)** Stepwise multivariate Cox regression analysis of CMRGs. The mRNA expressions of LOXL2 **(F)**, SLC31A2 **(G)** and SOD3 **(H)** in the LUAD and normal lung tissues. P values were shown as: *p < 0.05; **p < 0.01.

34 differentially expressed CMRGs were identified between 496 LUAD and 59 normal tissues (**Figure 1A**; **Table S2**), including 20 downregulated and 13 upregulated CMRGs (**Figure 1B**). Univariate Cox regression analysis showed that 11 CMRGs were associated with OS (P < 0.05, **Table S3**). Lasso Cox regression analysis further reduced candidate CMRGs to 9 (**Figures 1C, D**). Finally, 3 key CMRGs and their corresponding coefficients were determined by multivariate Cox regression analysis (**Figure 1E**). Risk score of each patient = LOXL2_exp_*0.228-SLC31A2 _exp_ *1.914-SOD3 _exp_ *0.173. The qRT-PCR results from 10 paired LUAD samples showed that the expression of SOD3 and SLC31A2 in cancer tissues was significantly lower than that in normal lung tissues, whereas LOXL2 has highly expression in LUAD tissues (P < 0.05, **Figures 1F-H**).

### Prognostic value and validation of CMRGS

**Figure 2:**
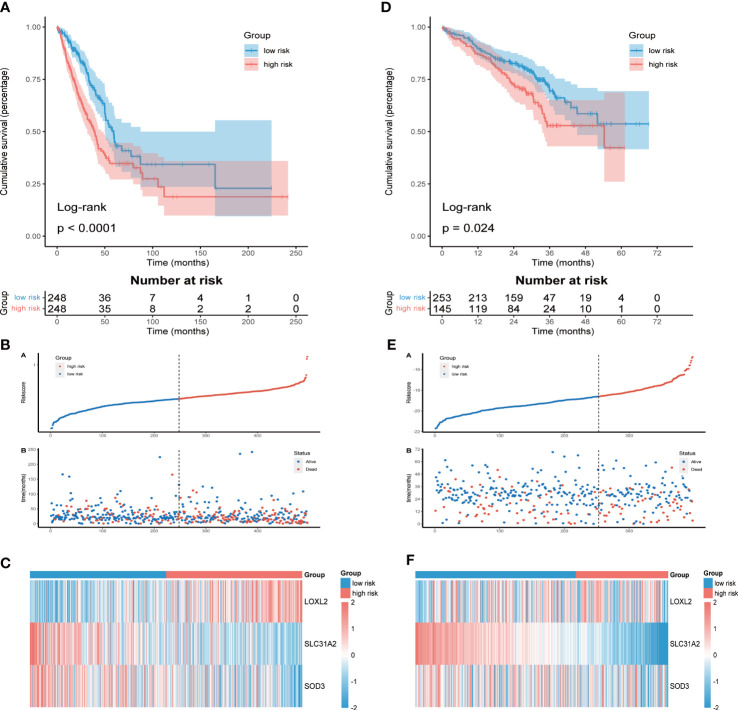
Assessment and validation of prognostic value of CMRGS. **(A)** Kaplan-Meier survival curves of OS in TCGA cohort. **(B)** Distribution of risk score and OS status in TCGA cohort. **(C)** Heatmap of the three CMRGs in TCGA. **(D)** Kaplan-Meier survival curves of OS in GSE72094. **(E)** Distribution of risk score and OS status in GSE72094. **(F)** Heatmap of the three CMRGs in GSE72094.

LUAD patients were divided into high-risk group (n = 248) and low-risk group (n = 248) according to the median risk score. KM survival analysis showed that OS of high-risk group was significantly shorter than that of low-risk group (P<0.0001, **Figure 2A**). The distribution of risk score and the survival status of each LUAD patient were shown in **Figure 2B**. The differential expression of LOXL2, SLC31A2 and SOD3 between the two groups was shown by heatmap (**Figure 2C**). Consistent results were also obtained in the validation cohort GSE72094 (**Figures 2D-F**). To verify the stability of CMRGS, we performed survival analysis on different subgroups. The results showed that the OS of low-risk group was significantly higher than that of high-risk group regardless of age, sex, T stage and N stage (p < 0.05, **Figure S2**). However, there was no significant difference in survival between M1 and stage III + IV groups, which may be due to the small sample size. These results indicated that CMRGS could be used as an effective predictor to evaluate the prognosis of LUAD patients.

### Coexpression of transcription factors and GO/KEGG enrichment analysis

**Figure 3:**
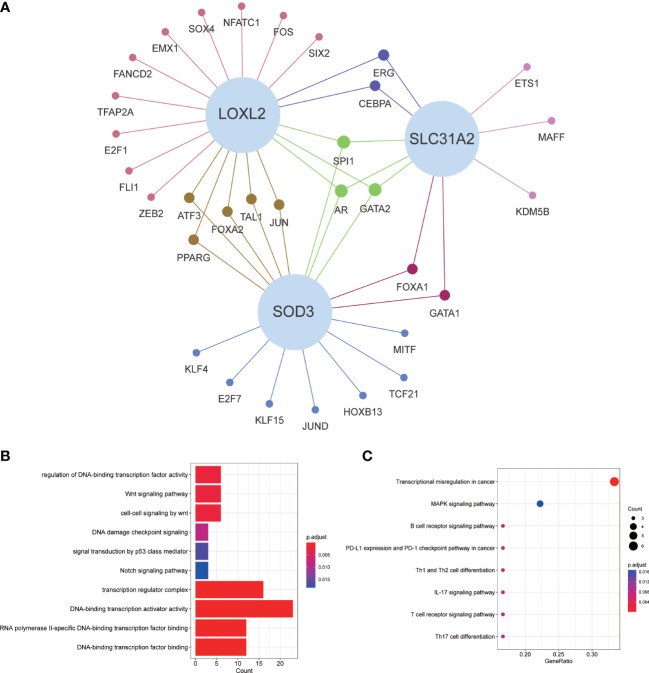
Transcriptional factor regulatory network and GO/KEGG enrichment analysis. **(A)** Regulatory network of key CMRGs and transcription factors. **(B)** GO enrichment analysis. **(C)** KEGG enrichment analysis.

For exploring the potential mechanisms and pathways of key CMRGs, we screened the differentially expressed transcription factors from Cistrome database, which were related to CMRGs. The relationship network between them was shown in **Figure 3A**. GO enrichment analysis results suggested that these transcription factors were closely associated with cancer-related pathways, such as p53, notch and wnt signaling pathway (**Figure 3B**). KEGG results showed that transcription factors were mainly related to multiple immune pathways (**Figure 3C**).

### Development of the nomogram

**Figure 4:**
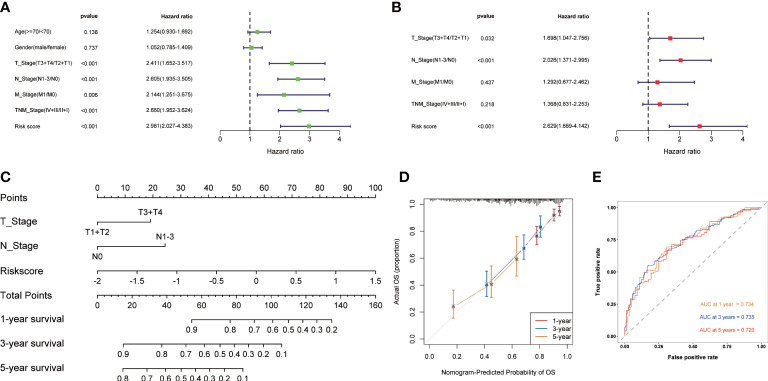
Construction of the nomogram based on CMRGS in TCGA cohort. Univariate **(A)** and multivariate **(B)** analysis of based on risk score and clinical features. **(C)** Nomogram for the prediction of 1-, 3- and 5-year survival probability. **(D)** Calibration curves for evaluating the agreement in the predicted and actual OS. **(E)** Time-dependent ROC analysis of the nomogram.

Univariate and multivariate Cox regression analysis were used to evaluate whether CMRGS was an independent risk factor for LUAD prognosis. In univariate Cox regression analysis, T stage, N stage, M stage, TNM stage and risk score were significantly correlated with OS (**Figure 4A**). In multivariate Cox regression analysis, T stage, N stage and risk score were still independent risk factors for prognosis (**Figure 4B**). Subsequently, the nomogram for predicting OS of LUAD patients at 1, 3, and 5 years were constructed based on T stage, N stage, and risk score (**Figure 4C**). The calibration curve showed that the predicted results of OS in 1, 3 and 5 years were consistent with the actual results (**Figure 4D**). The AUC of 1-, 3- and 5-year OS were 0.734, 0.735 and 0.720, respectively (**Figure 4E**). Taken together, these findings suggested that the nomogram could predict short-term or long-term OS of LUAD patients more accurately than a single prognostic factor.

### Association of TMB

**Figure 5:**
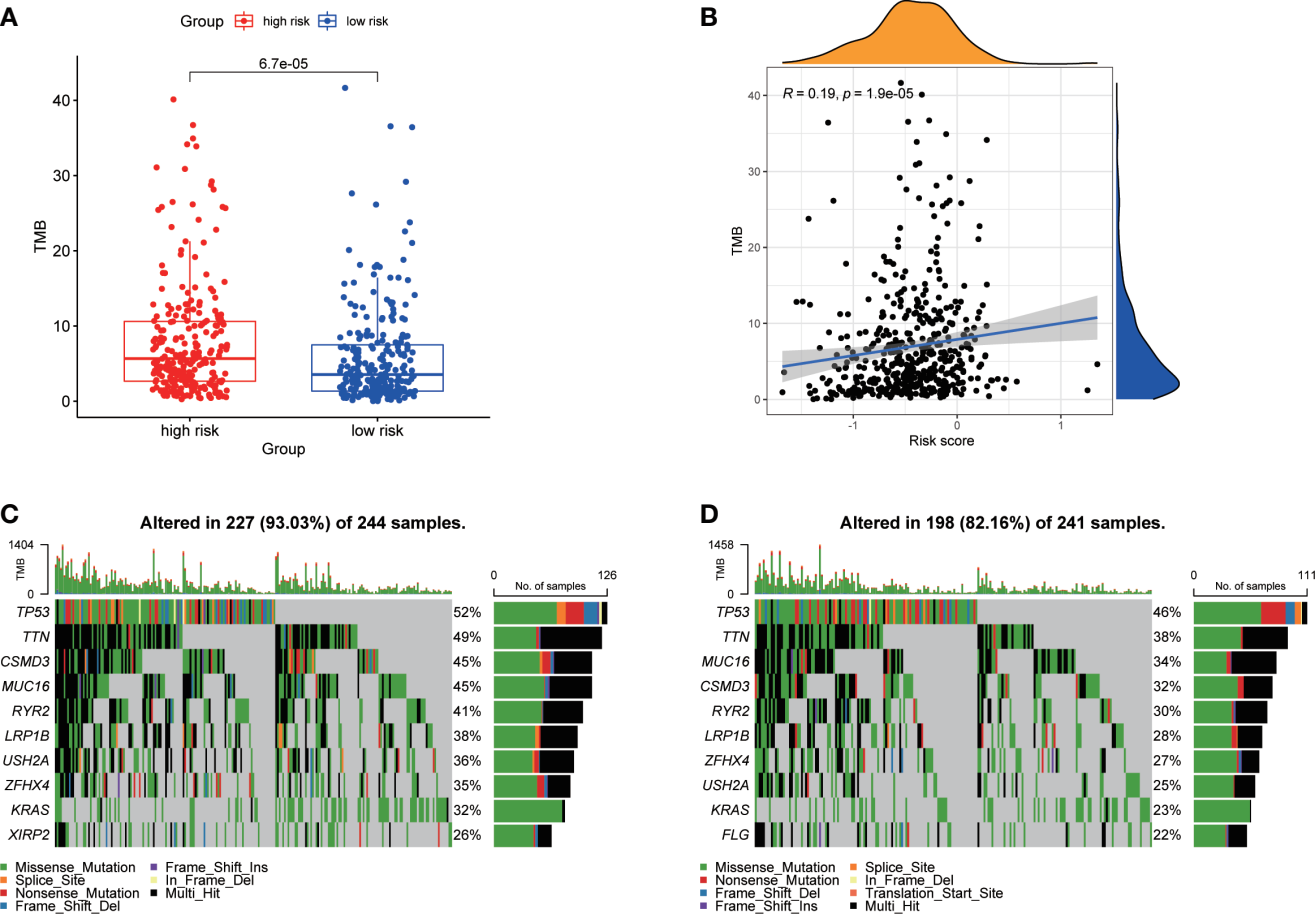
Characteristics of tumor mutation burden in TCGA cohort. **(A)** Differences of TMB between high- and low-risk groups. **(B)** Correlation analysis of risk score and TMB. **(C)** Top 10 genes with mutation frequency in high-risk group. **(D)** Top 10 genes with mutation frequency in low-risk group.

We calculated the TMB of each LUAD patient from TCGA. For the entire LUAD cohort, the most common top 10 mutations were TP53, TNN, MUC16, CSMD3, RYR2, LRP1B, ZFHX4, USH2A, KRAS and XIRP2 (**Figure S3A**). The TMB of high-risk group was significantly higher than that of low-risk group (**Figure 5A**), and the risk score was positively correlated with TMB (**Figure 5B**). The waterfall plots showed the mutation of the two groups (**Figures 5C, D**). The mutation frequency of the high-risk group was higher, and the detailed mutation statistics were shown in **Figures S3B, C**.

### Difference of biological function between two CMRGS groups

**Figure 6:**
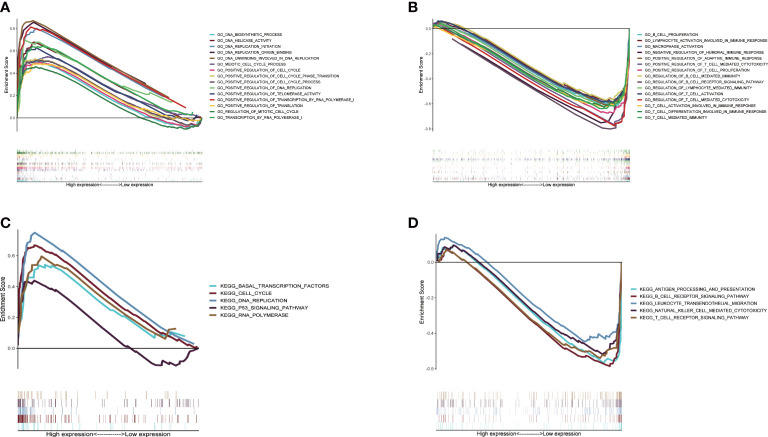
Gene set enrichment analysis between high- and low-risk groups based on LMRGS. Enrichment of GO in high-risk group **(A)** and low-risk group **(B)**. Enrichment of KEGG in high-risk group **(C)** and low-risk group **(D)**.

In order to study the molecular mechanism related to CMRGS based on three CMRGs in LUAD patients, we performed GSEA analysis on high-risk and low-risk groups. Go enrichment analysis found that the high-risk group was mainly related to DNA replication, DNA transcription and the regulation of cell cycle (**Figure 6A**). In the low-risk group, a large number of immune related pathways were activated (**Figure 6B**). The results of KEGG enrichment analysis also confirmed the above results (**Figures 6C, D**).

### Analysis of cell infiltration in TME

**Figure 7:**
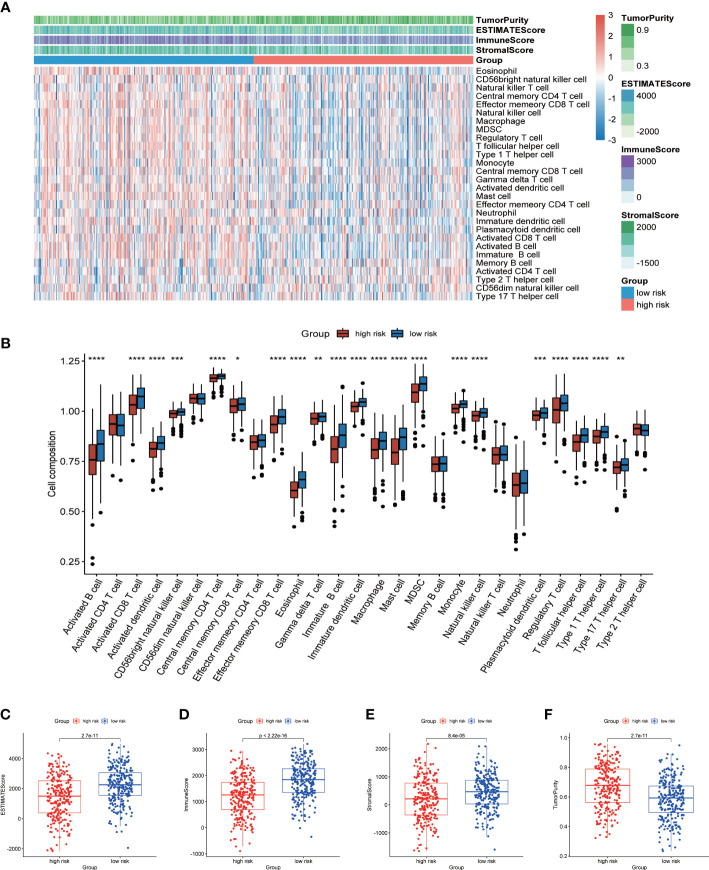
Analysis of TME cell infiltration. **(A)** Heatmap showing ssGSEA scores, ESTIMATE score, immune score, stromal score, and tumor purity between high- and low-risk groups. **(B)** The differences in the proportions of 28 immune cells between high- and low-risk groups. Differences of ESTIMATE score **(C)**, immune score **(D)**, stromal score **(E)** and tumor purity **(F)** in high- and low-risk groups. P values were shown as: *p < 0.05; **p < 0.01; ***p < 0.001; ****p < 0.0001.

In view of the enrichment of a large number of immune related pathways in the low-risk group, we then used ssGSEA to evaluate the difference in immune cell components between the high- and low-risk group. The heatmap showed the infiltration of 28 kinds of immune cells in all LUAD patients (**Figure 7A**). We found significant differences in the proportion of 21 types of immune cells (**Figure 7B**). More importantly, all of these 21 types of immune cells showed higher infiltration levels in the low-risk group. This also verified that a large number of immune related pathways were activated in the low-risk group. The results of ESTIMATE analysis showed that the ESTIMATE score, immune score and stromal score of the low-risk group were significantly higher than those of the high-risk group, and the tumor purity of the high-risk group was higher (**Figures 7C-F**). The above results suggested that TME was activated in the low-risk group, so the prognosis was better.

### Prediction of immunotherapy response based on CMRGS

**Figure 8:**
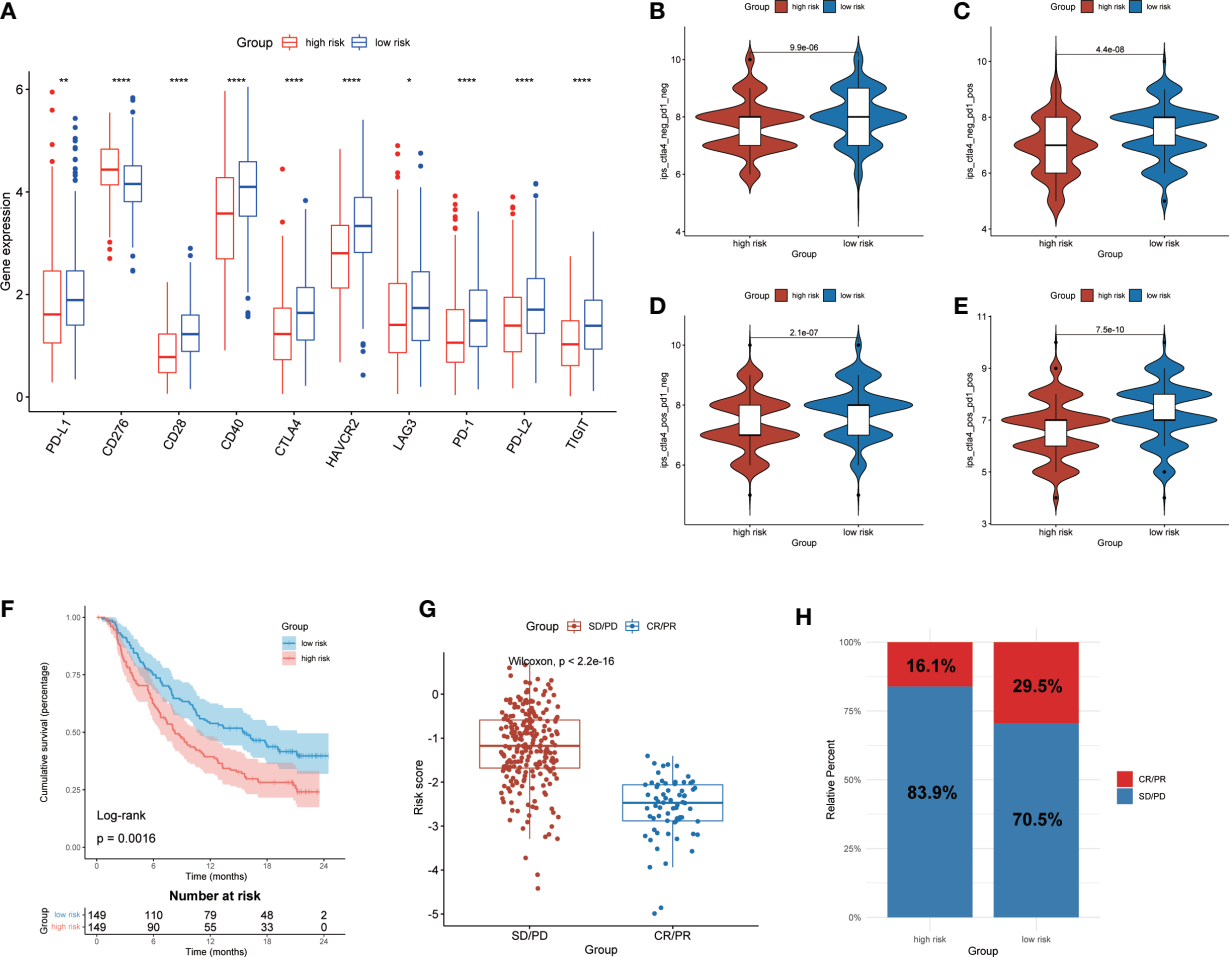
Prediction of immunotherapy response. **(A)** Expression of 10 immune checkpoints between high- and low-risk groups. Comparison of IPS in two groups. **(B)** CTLA4_negtive_/PD-1_negtive_. **(C)** CTLA4_negtive_/PD-1_positive_. **(D)** CTLA4_positive_/PD-1_negtive_. **(E)** CTLA4_positive_/PD-1_positive._ **(F)** Kaplan–Meier analysis of OS by CMRG signature for patients in the IMvigor210 cohort (P=0.0016). **(G)** Difference of risk score between different response groups. **(H)** Proportions of anti-PD-L1 immunotherapy response in high- and low-risk groups. CR, Complete Response, PR, Partial Response, SD, Stable Disease, and PD, Progressive Disease. P values were shown as: *p < 0.05; **p < 0.01; ****p < 0.0001.

In order to further investigate the potential correlation between CMRGS and immunotherapy, we compared the expression differences of 10 key immune checkpoints (PD-L1, CD276, CD28, CD40, CTLA-4, HAVCR2, LAG3, PD-1, PD-L2 and TIGIT) between the two groups. The results showed that there were significant differences in the expression of these 10 immune checkpoints. Except for CD276, the other 9 immune checkpoints had higher expression levels in the low-risk group (**Figure 8A**). IPS was used to evaluate the therapeutic efficacy of immune checkpoint inhibitors, and the results showed that the efficacy of low-risk group was significantly better than that of high-risk group regardless of the status of CTLA-4 and PD-1 (**Figures 8B-E**). In addition, we also utilized the IMvigor210 cohort of anti-PD-L1 immunotherapy to predict the response to immunotherapy. The risk score of 298 samples was calculated by the above formula, and divided into two groups according to the median risk score. Low-risk group exhibits a better prognosis (P=0.0016, **Figure 8F**). Furthermore, risk score in different response groups had significant difference (**Figure 8G**), and the proportion of CR/PR was higher in the low-risk group of patients (**Figure 8H**). The efficacy of anti-PD-L1 immunotherapy was closely related to the expression of PD-L1, thus we analyzed the relationship between risk score and IC, TC and immune phenotype. The results showed that lower risk score was associated with higher IC expression (**Figures S4A, B**). There was no significant difference in risk score between different TC groups (**Figures S4C, D**). In addition, immune-inflamed type had the lower risk score than the immune-desert and the immune-excluded type (**Figures S4E, F**). These results showed that CMRGS could evaluate the efficacy of immunotherapy, and patients with lower risk score will better benefit from immunotherapy.

## Discussion

Copper is an essential micronutrient and participates in a variety of physiological processes. Previous research implying that reprogramming of copper metabolism interferes with cancer cell proliferation and invasion (26, 27). Currently, therapeutic strategies targeting copper or copper metabolizing proteins have also been developed (28, 29). Given the important role of copper in cancer, this study aims to explore the role of CMRGs in LUAD.

In this study, we identified the prognosis-related CMRGS based on differentially expressed CMRGs, including LOXL2, SLC31A2 and SOD3. LOXL2 is a copper dependent amine oxidase involved in tumor invasion and metastasis. Copper ion is an indispensable cofactor of the enzyme activity. The increase of copper content can significantly activate LOXL2 (30). Free copper ions are toxic to cells. SLC31A2 (also known as copper transporter 2) promotes copper absorption and regulates copper homeostasis. A large amount of copper accumulation was detected in SLC31A2 knockout mice (31). Excessive copper will produce significant cytotoxicity to cells. In our study, the expression of SLC31A2 is lower in high-risk patients, which may be one of the reasons for their poor prognosis. SOD3 is a Cu-containing secretory enzymes, and its reduced content will lead to oxidative stress and epithelial-mesenchymal transformation, thereby enhancing tumor progression (32). In addition, overexpression of SOD3 can enhance the infiltration of CD4+ and CD8+ T cells in the TME (33). Thus, the efficacy of immunotherapy may be increased.

Based on the expression of the 3 CMRGs, LUAD patients were divided into high- and low-risk groups. The prognosis of the low-risk group was significantly better than that of the high-risk group. Multivariate cox regression analysis confirmed that CMRGS was an independent risk factor for LUAD. Subsequently, in order to facilitate clinical application, we constructed a nomogram by combining clinical features to provide clinicians with an individualized scoring system.

To investigate the potential molecular functions of CMRGS, we performed GSEA analysis on different risk groups. Surprisingly, a large number of immune related pathways were enriched in the low-risk group. Previous study had shown that reducing the level of copper in tumor cells with copper chelating would increase the infiltration of CD8+ T cells and NK cells, and inhibit tumor growth (34). Therefore, we speculated that copper metabolism was closed to anti-tumor immunity. Then we analyzed the difference in TME between two groups. It was found that up to 21 types of immune cells were significantly infiltrated in the low-risk group. This confirmed our conjecture. More importantly, higher levels of immune cell infiltration represented better prognosis.

Since the expression level of immune checkpoints is a biomarker for predicting immunotherapy response (35). We also investigated differences in expression of 10 immune checkpoints between two groups. It was found that multiple immune checkpoints were significantly expressed in the low-risk group, including PD-L1, PD-1, CTLA-4, CD28, CD40, HAVCR2, LAG3, PD-L2 and TIGIT. This suggested that patients in the low-risk group may benefit better from immunotherapy. Subsequently, we used IPS and IMvigor210 cohort to predict the response to immunotherapy in the two groups. As expected, the low-risk group exhibited better prognosis and immunotherapy response due to the immune activation of the TME. The above results suggested that CMRGS could guide immunotherapy in patients with LUAD.

There are some limitations in this study. First, the construction and validation of CMRGS is based on data from public databases. It needs to be further validated by multicenter and prospective studies in the future. Second, further experiments are needed to verify the individual or combined roles of the 3 genes involved in CMRGS in LUAD.

## Conclusion

CMRGS is a promising biomarker to assess the prognosis of LUAD patients and may be serve as a guidance on immunotherapy.

## Data availability statement

The datasets presented in this study can be found in online repositories. The names of the repository/repositories and accession number(s) can be found in the article/**Supplementary Material**.

## Ethics statement

The studies involving human participants were reviewed and approved by Sun Yat-sen University Cancer Center ethics committee (YB2018-85). The patients/participants provided their written informed consent to participate in this study.

## Author contributions

WC and HL, conceptualization, data curation, formal analysis, writing–original draft, writing–review and editing. LZ, TZ, ZC, and WO, data-collecting, writing–review and editing. SW, conceptualization, supervision, funding acquisition, writing–original draft, project administration, writing–review and editing. All authors contributed to the article and approved the submitted version.

## Funding

This work was supported by the National Collaborative Project for Major and Intractable Diseases of China (KY022102).

## Acknowledgments

We acknowledge TCGA and GEO database for providing their platforms and contributors for uploading their meaningful datasets.

## Conflict of interest

The authors declare that the research was conducted in the absence of any commercial or financial relationships that could be construed as a potential conflict of interest.

## Publisher’s note

All claims expressed in this article are solely those of the authors and do not necessarily represent those of their affiliated organizations, or those of the publisher, the editors and the reviewers. Any product that may be evaluated in this article, or claim that may be made by its manufacturer, is not guaranteed or endorsed by the publisher.

## Supplementary material

Supplementary Table 1 Copper metabolism-related genes.

Supplementary Table 2 Differentially expressed CMRGs.

Supplementary Table 3 Univariate Cox regression analysis of differentially expressed CMRGs.

Supplementary Figure 1 Study design and flowchart.

Supplementary Figure 2 Stratified survival analysis based on CMRGS. **(A)** Age<70 and Age>=70. **(B)** Female and male. **(C)** T1+T2 and T3+T4. **(D)** N0 and N1+N2+N3. **(E)** M0 and M1. **(F)** Stage I+II and Stage III+IV.

Supplementary Figure 3 Genetic alterations of LUAD in TCGA **(A)** Mutation of all LUAD in TCGA. **(B)** Mutation statistics in high-risk group. **(C)** Mutation statistics in low-risk group.

Supplementary Figure 4 Evaluation of response to immunotherapy. Difference of risk score in different IC expression **(A)**, TC expression **(B)** and immune phenotype **(C)** in IMvigor210 cohort.
