# Supplementary figures and images for: Development of a copper metabolism-related gene signature in lung adenocarcinoma
Source: Front Immunol. 2022 Nov 29;13:1040668. doi: 10.3389/fimmu.2022.1040668 (PMC9744782; doi:10.3389/fimmu.2022.1040668)

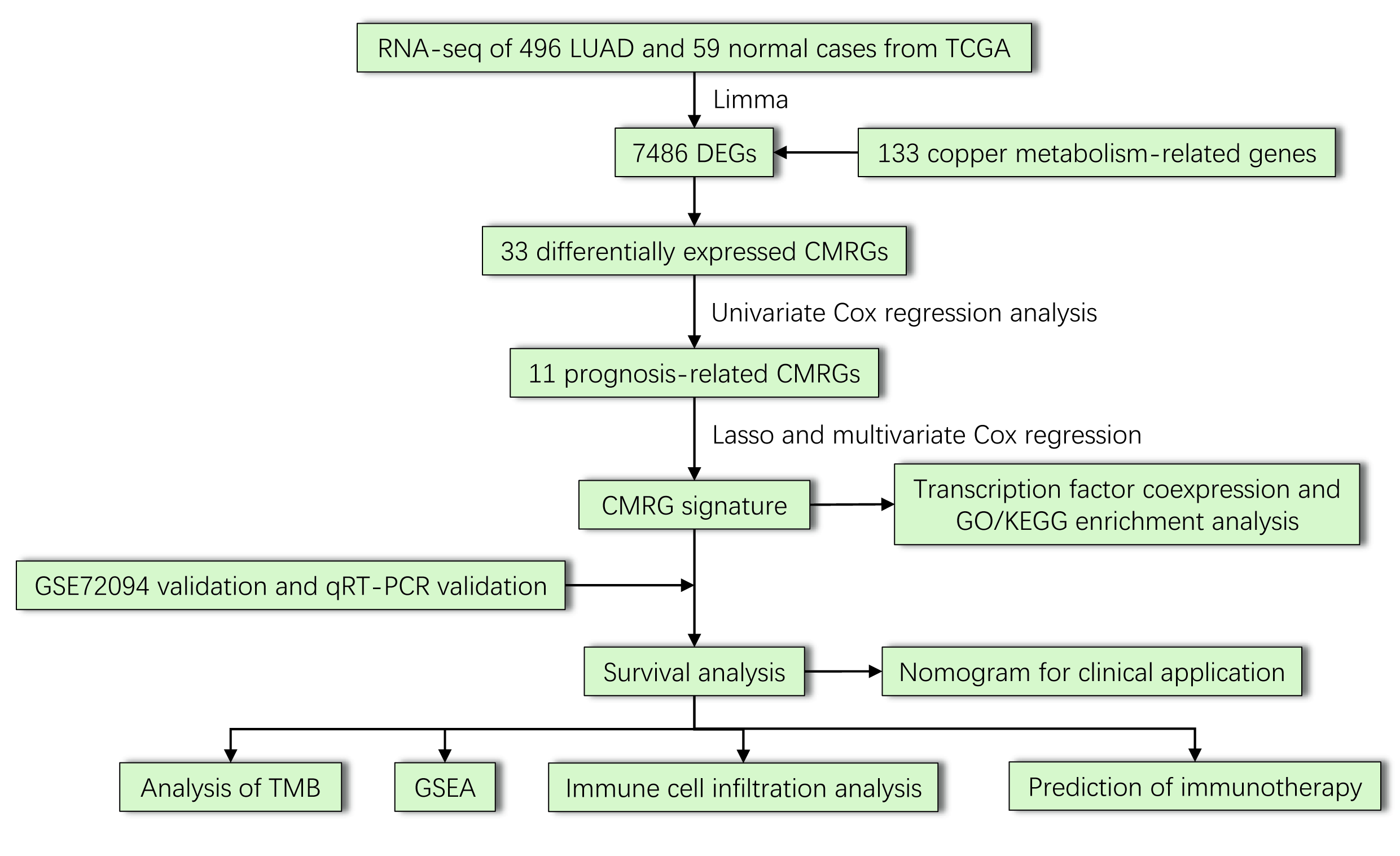

Supplement: Supplementary Figure 1 — Study design and flowchart. [file Image_1.tif]

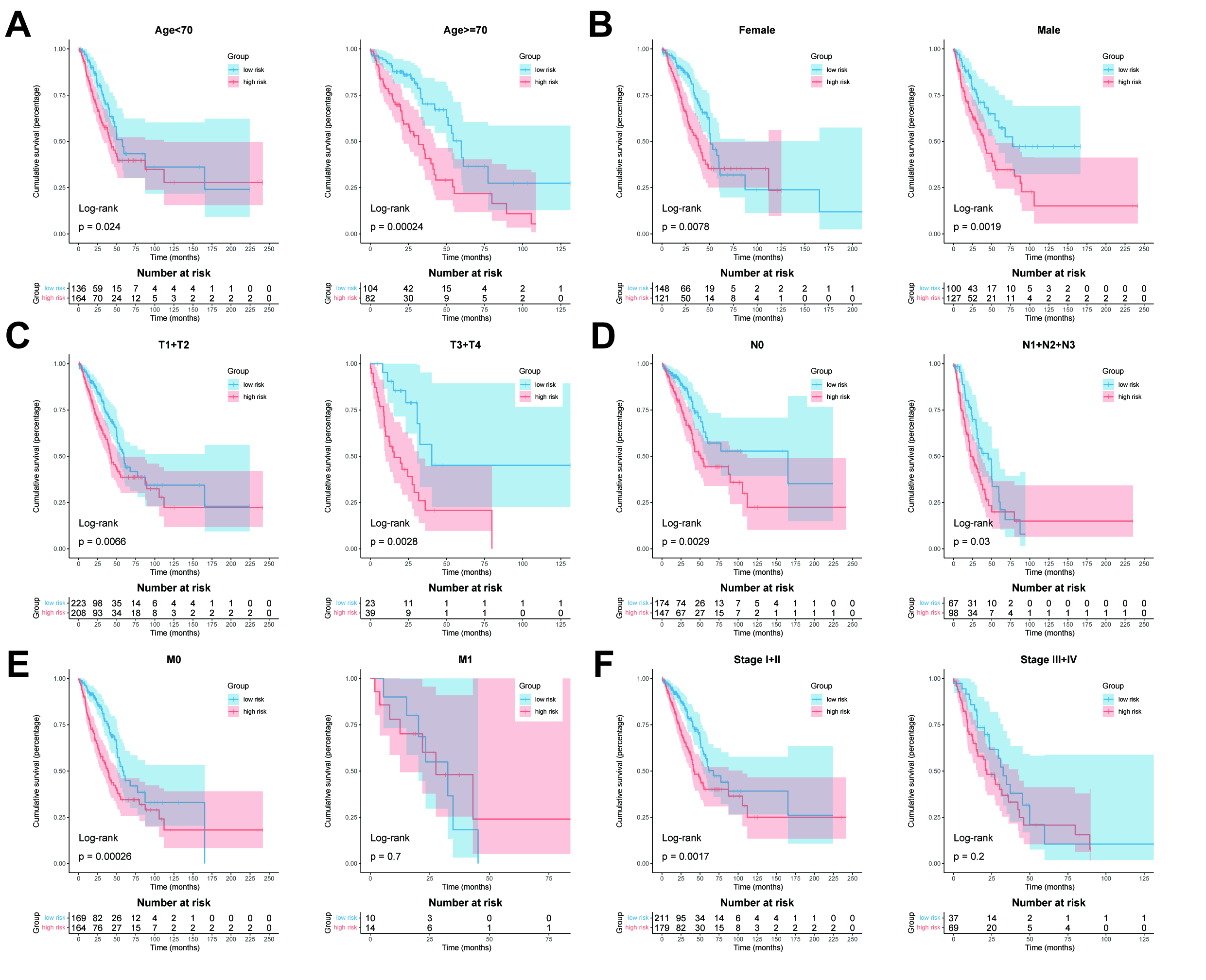

Supplement: Supplementary Figure 2 — Stratified survival analysis based on CMRGS. (A) Age<70 and Age>=70. (B) Female and male. (C) T1+T2 and T3+T4. (D) N0 and N1+N2+N3. (E) M0 and M1. (F) Stage I+II and Stage III+IV. [file Image_2.tif]

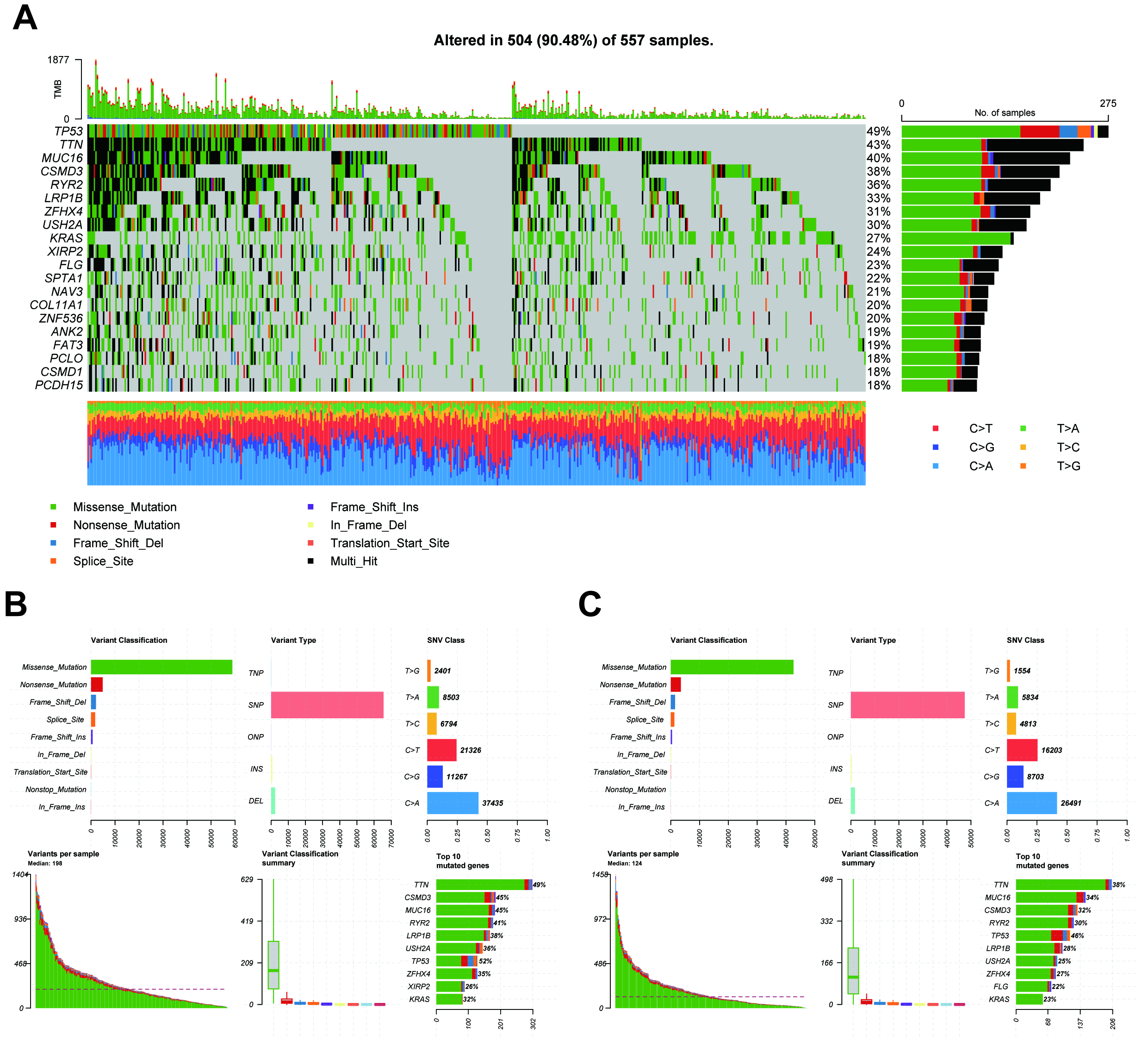

Supplement: Supplementary Figure 3 — Genetic alterations of LUAD in TCGA (A) Mutation of all LUAD in TCGA. (B) Mutation statistics in high-risk group. (C) Mutation statistics in low-risk group. [file Image_3.tif]

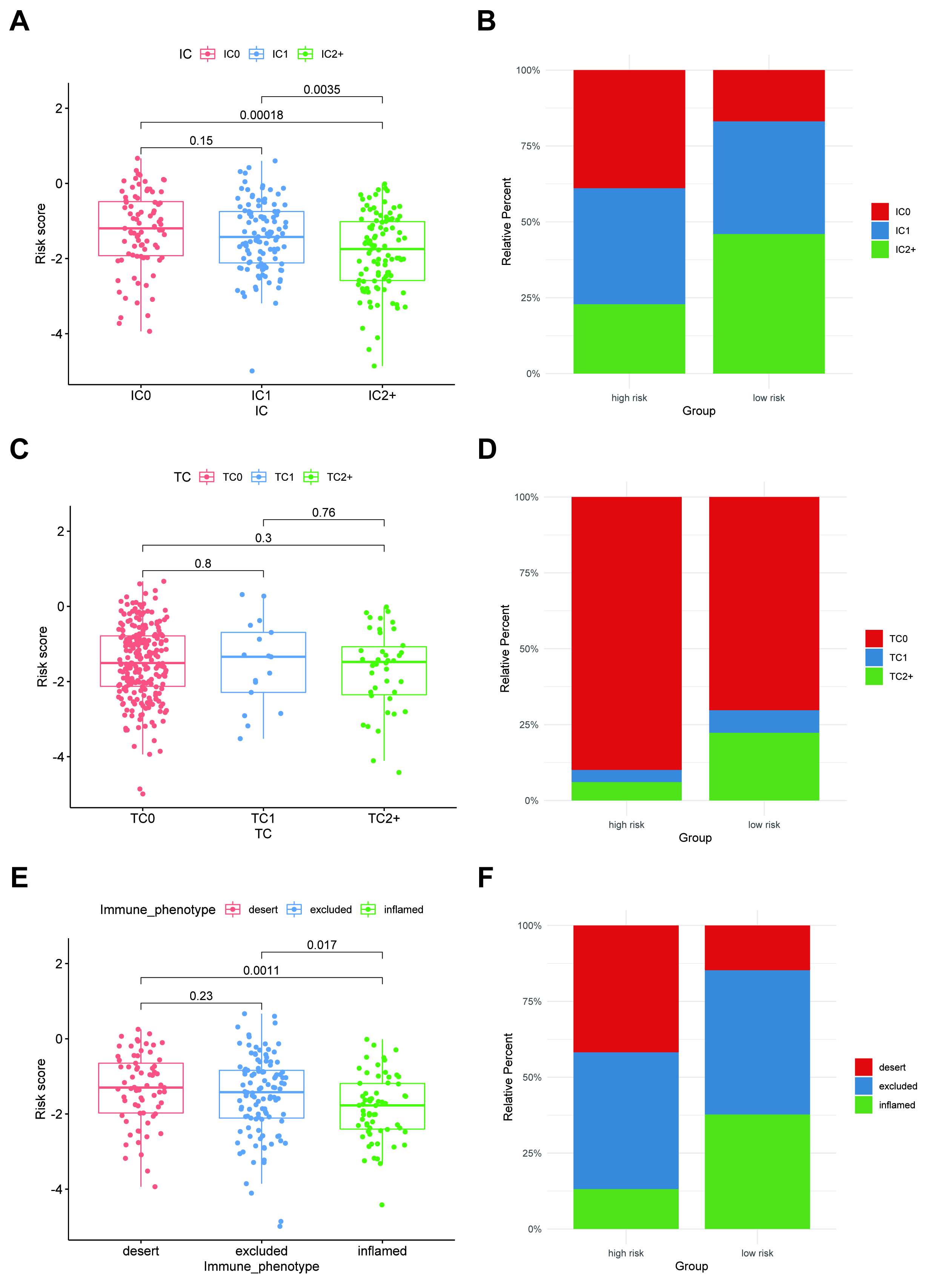

Supplement: Supplementary Figure 4 — Evaluation of response to immunotherapy. Difference of risk score in different IC expression (A), TC expression (B) and immune phenotype (C) in IMvigor210 cohort. [file Image_4.tif]
